# Potential pharmacological mechanisms of four active compounds of *Macleaya cordata* extract against enteritis based on network pharmacology and molecular docking technology

**DOI:** 10.3389/fphys.2023.1175227

**Published:** 2023-05-02

**Authors:** Pingrui Yang, Chonghua Zhong, Huan Huang, Xifeng Li, Lin Du, Lifang Zhang, Shicheng Bi, Hongxu Du, Qi Ma, Liting Cao

**Affiliations:** ^1^ Department of Traditional Chinese Veterinary Medicine, College of Veterinary Medicine, Southwest University, Chongqing, China; ^2^ College of Animal Science and Technology, Southwest University, Chongqing, China; ^3^ College of Animal Science and Technology, Yunnan Agricultural University, Kunming, China; ^4^ Chi Institute of Traditional Chinese Veterinary Medicine, Southwest University, Chongqing, China

**Keywords:** *Macleaya cordata* extract, enteritis, network pharmacology, molecular docking, mechanism

## Abstract

**Background:** *Macleaya cordata* extract (MCE) is effective in the treatment of enteritis, but its mechanism has not been fully elucidated. Therefore, this study combined network pharmacology and molecular docking technologies to investigate the potential pharmacological mechanism of MCE in the treatment of enteritis.

**Methods:** The information of active compounds in MCE was accessed through the literature. Furthermore, PubChem, PharmMapper, UniProt, and GeneCards databases were used to analyze the targets of MCE and enteritis. The intersection of drug and disease targets was imported into the STRING database, and the analysis results were imported into Cytoscape 3.7.1 software to construct a protein–protein interaction (PPI) network and to screen core targets. The Metascape database was used for conducting Gene Ontology (GO) enrichment and Kyoto Encyclopedia of Genes and Genomes (KEGG) pathway analyses. AutoDock Tools software was used for the molecular docking of active compounds with the core targets.

**Results:** MCE has four active compounds, namely, sanguinarine, chelerythrine, protopine, and allocryptopine, and a total of 269 targets after de-duplication. Furthermore, a total of 1,237 targets were associated with enteritis, 70 of which were obtained by aiding the drug–disease intersection with the aforementioned four active compound targets of MCE. Five core targets including mitogen-activated protein kinase 1 (MAPK1) and AKT serine/threonine kinase 1 (AKT1) were obtained using the PPI network, which are considered the potential targets for the four active compounds of MCE in the treatment of enteritis. The GO enrichment analysis involved 749 biological processes, 47 cellular components, and 64 molecular functions. The KEGG pathway enrichment analysis revealed 142 pathways involved in the treatment of enteritis by the four active compounds of MCE, among which PI3K-Akt and MAPK signaling pathways were the most important pathways. The results of molecular docking showed that the four active compounds demonstrated good binding properties at the five core targets.

**Conclusion:** The pharmacological effects of the four active compounds of MCE in the treatment of enteritis involve acting on signaling pathways such as PI3K-Akt and MAPK through key targets such as AKT1 and MAPK1, thus providing new indications for further research to verify its mechanisms.

## 1 Introduction

Enteritis, a common intestinal disease that can be caused by viruses, bacteria, fungi, or parasites, has the potential to compromise human health (Ryan et al., 2018; Leon-Velarde et al., 2019; Dacal et al., 2020; Lim et al., 2020; Dixon, 2021; Tarris et al., 2021). When the human body is unable to balance the intestinal microflora and pathogenic stimuli, enteritis occurs. In a dysfunctional immune system, the lack of such a balance causes an increase in the inflammatory response (Moreira Lopes et al., 2020). Clinical symptoms include abdominal pain, diarrhea, fever, and even death in severe cases (Cleary, 1998). Chinese herbal medicine is edible and has remarkable medicinal effects, with few residues and fewer side effects. *Macleaya cordata* is a perennial, erect, and tall herb of the poppy family, with the advantages of clearing away heat, detoxification, subduing swelling, and eliminating parasites. *Macleaya cordata* extract (MCE) has anti-inflammatory, antibacterial, and anti-tumor effects (Lin et al., 2018). Soler-Vasco et al. (2016) reported that MCE helps reduce the inflammatory responses of intestinal epithelial cells caused by enterotoxigenic *Escherichia coli*.

Network pharmacology, based on systems biology and network equilibrium theory, combines pharmacology and database application systems, computational chemistry and other tools to directly identify drugs and disease targets from a large amount of data and explain the disease development process and the drug–organism interaction mechanism (Hao and Xiao, 2014; Liu et al., 2015; Zhou et al., 2020). This technique can be further combined with the holistic concept of traditional Chinese medicine (TCM) and evidence-based treatment concept to systematically analyze the mechanism of drug action in the body (Zhang et al., 2019; Wu et al., 2021; Jiashuo et al., 2022). It is well known that the chemical composition of Chinese herbal medicine is complex, whether single or herbal compounds that are, in general, multiple components acting on multiple targets and multiple pathways (Zhang et al., 2017; Yuan et al., 2022). This property leads to difficulties in elucidating the mechanisms of Chinese herbal medicine at the cellular and molecular levels *in vivo* (Zhang et al., 2017). The interaction between a disease and biomolecule can be determined by combining network pharmacology and TCM. Molecular docking techniques are used to study the binding sites and interactions between small molecules of drugs and target macromolecules, and these can also be used to simulate the affinity process and calculate the binding energy between the two, thus improving the efficiency and relevance of Chinese herbal medicine screening (Pagadala et al., 2017; Han et al., 2018; Salmaso and Moro, 2018; Jiao et al., 2021). So network pharmacology and molecular docking technologies were used in this study to predict and verify the core targets and signaling pathways of the four active compounds of MCE in the treatment of enteritis and build a multi-interaction network of “drug–active compound–target–pathway–disease.” The mechanism of the four active compounds of MCE in the treatment of enteritis was comprehensively and systematically analyzed to provide a theoretical basis for an in-depth study of their roles in the prevention and treatment of enteritis.

## 2 Materials and methods

### 2.1 Acquisition of active compounds and targets of MCE

Literature search engines such as China National Knowledge Infrastructure (https://www.cnki.net/) and PubMed (https://pubmed.ncbi.nlm.nih.gov/) were used to review the main active substances of MCE and their active compounds. The 2D structures of the active compounds were consulted and downloaded from the PubChem database (https://pubchem.ncbi.nlm.nih.gov/). Then, the 2D structures were imported into the PharmMapper database (http://www.lilab-ecust.cn/pharmmapper/) to obtain UniPlot (Li et al., 2019). Furthermore, protein encodings were converted to gene names using the UniProt database (https://www.uniprot.org/) to obtain the targets for the active compounds.

### 2.2 Acquisition of enteritis-related targets

The GeneCards database (https://www.genecards.org/) was searched using “enteritis” as the keyword, and the results were exported in an Excel format. The data were de-duplicated and filtered based on the relevance score of >1, and the resulting data were considered enteritis-related targets.

### 2.3 Acquisition of intersection targets of active compounds and enteritis

The active compound targets of MCE obtained in Section 2.1 and the targets of enteritis obtained in Section 2.2 were imported into List2 and List1 of the Venny 2.1.0 online website (https://bioinfogp.cnb.csic.es/tools/venny/), respectively, to obtain the drug–disease intersection target Venn diagrams. The intersection site in the diagram was clicked to obtain the intersection target in the result table, which gave the potential target of MCE for the treatment of enteritis.

### 2.4 PPI network construction and analysis

The intersection targets were imported into the STRING database (https://cn.stringdb.org/) and set as “*Homo sapiens*” with a confidence level of 0.900, hidden unassociated proteins and protein–protein interaction (PPI) data were exported. PPI data were viewed using Cytoscape 3.7.1 software to build the PPI network graphs. The core targets were screened with the topological analysis based on the following criteria: degree ≥2* degree mean, betweenness centrality ≥betweenness centrality mean, and closeness centrality ≥closeness centrality mean. The higher the values of these three criteria, the more important the target was in the network (Xu et al., 2022).

### 2.5 GO enrichment and KEGG pathway analyses

The PPI data were de-duplicated and imported into the Metascape database (https://metascape.org/), and “*Homo sapiens*” was used as a species for Gene Ontology (GO) enrichment and Kyoto Encyclopedia of Genes and Genomes (KEGG) pathway analyses, which were arranged in the descending order of significance, −Log*P*. The top 10 enriched biological processes (BP), cellular components (CC), and molecular functions (MF) under the GO classification and the top 20 enriched KEGG pathways were then imported into Bioinformatics (http://www.bioinformatics.com.cn/) for visualization.

### 2.6 Construction of the drug–active compound–target–pathway–disease network

The drug, active compounds, intersection targets, pathway, and disease were imported into Cytoscape 3.7.1 software to construct the “drug–active compound–target–pathway–disease” network diagram and analyze the relationship among them. The more the number of connection nodes, the more the number of biological functions the nodes were involved in and the more their importance in the network (Gong et al., 2021).

### 2.7 Molecular docking verification

The 3D structures of the active compounds and core targets were downloaded from the PubChem and PDB databases (https://www.rcsb.org/), respectively. The SDF files of the active compounds were converted to PDB files using Open Babel GUI software. PyMOL software was used to dehydrate and desolvate the core targets. AutoDock Tools software was used to hydrogenate the active compounds and set them as the ligand and to hydrogenate the core targets and set them as the receptor. Molecular docking of the receptor and ligand was performed using AutoDock Tools software, and the docking result with the lowest binding energy was selected and imported into PyMOL software for visual analysis.

## 3 Results

### 3.1 Screening of active compounds in MCE and their targets

The literature review revealed that alkaloids are the main active substances in MCE (Zeng et al., 2013; Ni et al., 2016; Lin et al., 2018). Furthermore, the active compounds are sanguinarine (SAN), chelerythrine (CHE), protopine (PRO), and allocryptopine (ALL) (Table 1), yielding 200, 229, 171, and 156 targets, respectively, and yielding 269 targets after de-weighting (Supplementary Table S1).

**TABLE 1:** Information on the active compounds of *Macleaya cordata* extract (MCE).

Name	Molecular formula	Molecular weight	PubChem CID	2D structure
Sanguinarine	C_20_H_14_NO_4_^+^	332.3	5154	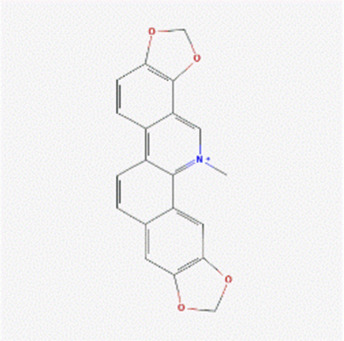
Chelerythrine	C_21_H_18_NO_4_^+^	348.4	2703	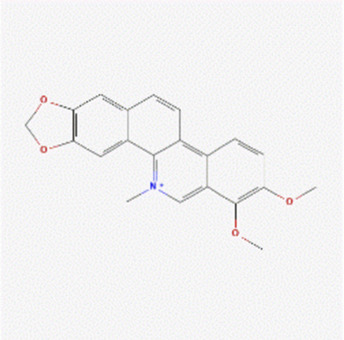
Protopine	C_20_H_19_NO_5_	353.4	4970	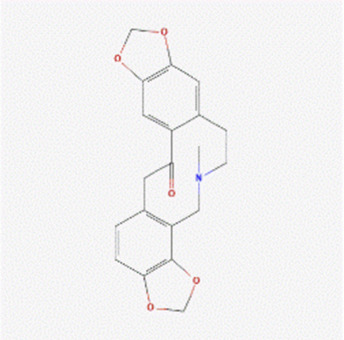
Allocryptopine	C_21_H_23_NO_5_	369.4	98570	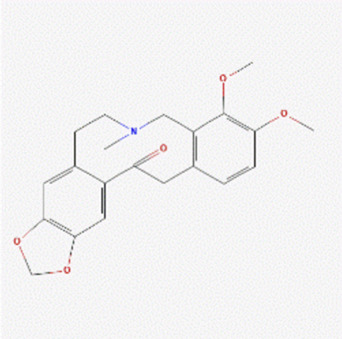

### 3.2 Enteritis-related target acquisition

From the GeneCards database, 9,027 enteritis-related targets were retrieved, and using de-duplication and screening based on the relevance score of >1, 1,237 enteritis-related targets were obtained, including actin gamma 2 (ACTG2), smooth muscle, RET proto-oncogene (RET), endothelin receptor type B (EDNRB), endothelin 3 (EDN3), SRY-box transcription factor 10 (SOX10), nucleotide-binding oligomerization domain-containing 2 (NOD2), glial cell-derived neurotrophic factor (GDNF), interleukin-6 (IL-6), tetratricopeptide repeat domain 37 (TTC37), and transthyretin (TTR) (Supplementary Table S2).

### 3.3 Intersection target of active compounds and enteritis

**FIGURE 1:**
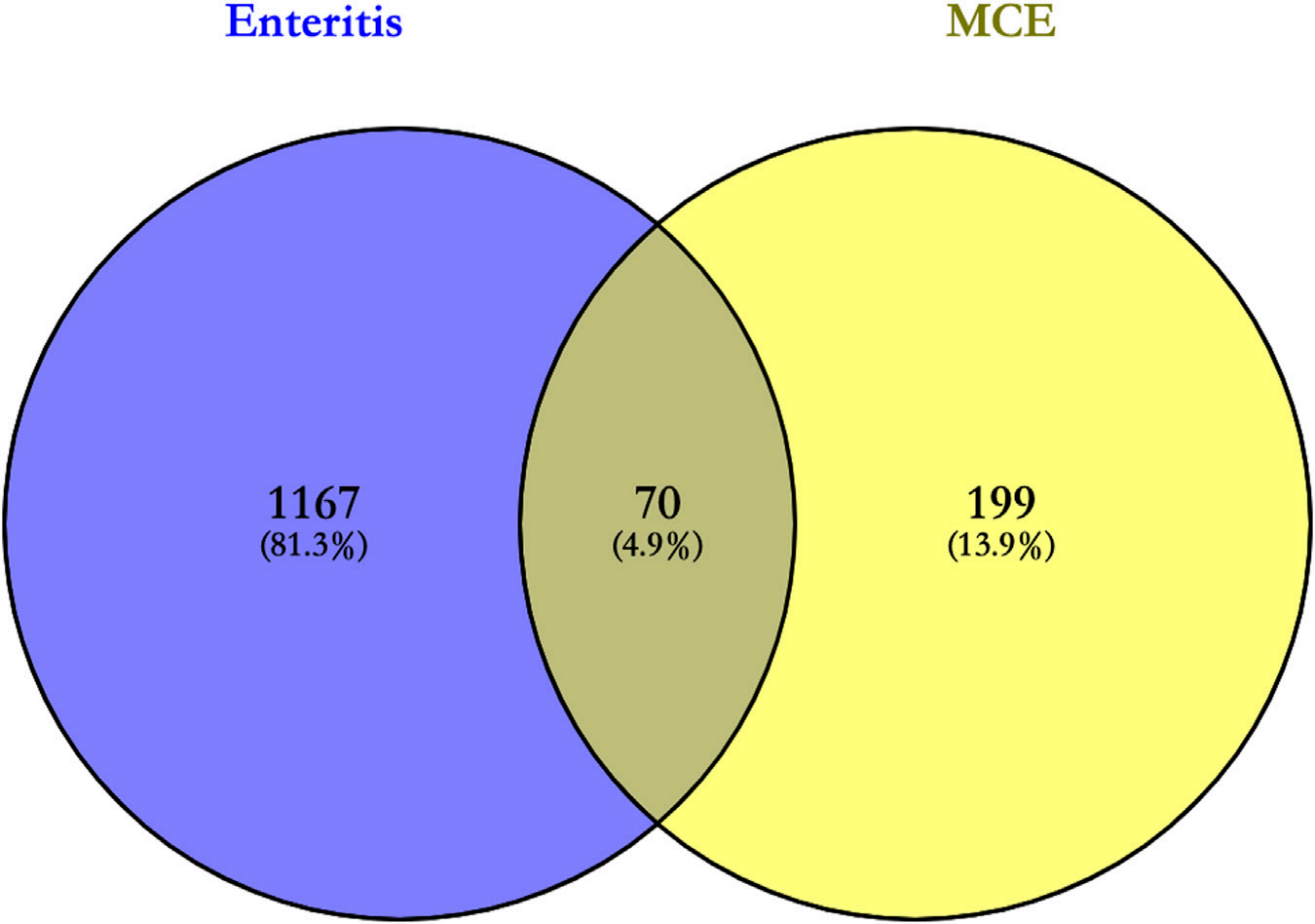
Venn diagram for the intersection of targets of enteritis and the four active compounds of MCE. The purple color on the left is the enteritis target, and the yellow color on the right is the target of the four active compounds of MCE; their intersection represents potential targets for the pharmacological effects of the four active compounds of MCE in the treatment of enteritis.

In total, 269 targets of the four active compounds of MCE and 1,237 targets of enteritis obtained in Section 3.1 and Section 3.2 were imported into Venny 2.1.0 for constructing Venn diagrams. Furthermore, 70 intersection targets were obtained, which were potential targets for the pharmacological effects of the four active compounds of MCE in the treatment of enteritis, including transthyretin (TTR), albumin (ALB), protein kinase C theta type (PRKCQ), interleukin-2 (IL-2), mast/stem cell growth factor receptor kit (KIT), insulin-like growth factor-I (IGF-1), peroxisome proliferator-activated receptor gamma (PPARG), tyrosine-protein kinase (BTK), vitamin D3 receptor (VDR), and retinol-binding protein 4 (RBP4) (Supplementary Table S3), which accounts for 4.9% of all targets (Figure 1).

### 3.4 PPI network construction and analysis results

**FIGURE 2:**
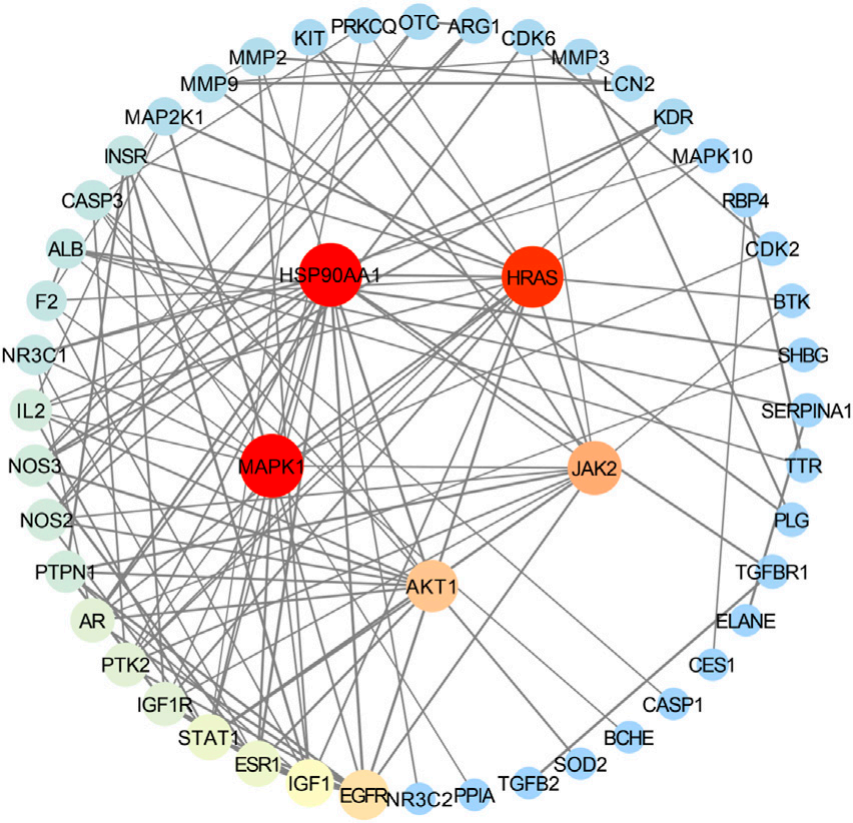
Protein–protein interaction (PPI) network diagram of the intersection target of enteritis and the four active compounds of MCE. Nodes represent targets, and the size and color are proportional to the degree value; the higher the degree value, the larger the node, which is indicated with colors from blue to red. Edges represent the association between targets, and thickness is related to the combination score. The thicker the line, the higher the binding between targets.

Using the STRING database, the highest confidence level (0.900) was selected in the minimum required interaction score column, while other conditions remained unchanged; we exported the Excel table and obtained 49 targets after de-weighting. After importing the 49 targets into Cytoscape 3.7.1 software, a PPI network diagram of enteritis and the four active compounds of targets of MCE (Figure 2) was obtained, with 49 nodes and 120 connections. In addition, topological analysis of these 49 nodes was performed using the plugin CytoNCA. According to the requirements, degree ≥ 9.795918367, betweenness centrality ≥0.044380518, and closeness centrality ≥0.343112989, the core targets were screened, including heat-shock protein 90 alpha family class A member 1 (HSP90AA1), mitogen-activated protein kinase 1 (MAPK1), HRas proto-oncogene, GTPase (HRAS), Janus kinase 2 (JAK2), and AKT serine/threonine kinase 1 (AKT1) (Table 2).

**TABLE 2:** Top five target information of the PPI network.

Name	Degree	Betweenness centrality	Closeness centrality
HSP90AA1	17	0.29526831	0.48484848
MAPK1	17	0.20624955	0.48
HRAS	16	0.15584066	0.47524752
JAK2	12	0.06196187	0.43243243
AKT1	11	0.09346604	0.42105263

### 3.5 Enrichment analysis results

**FIGURE 3:**
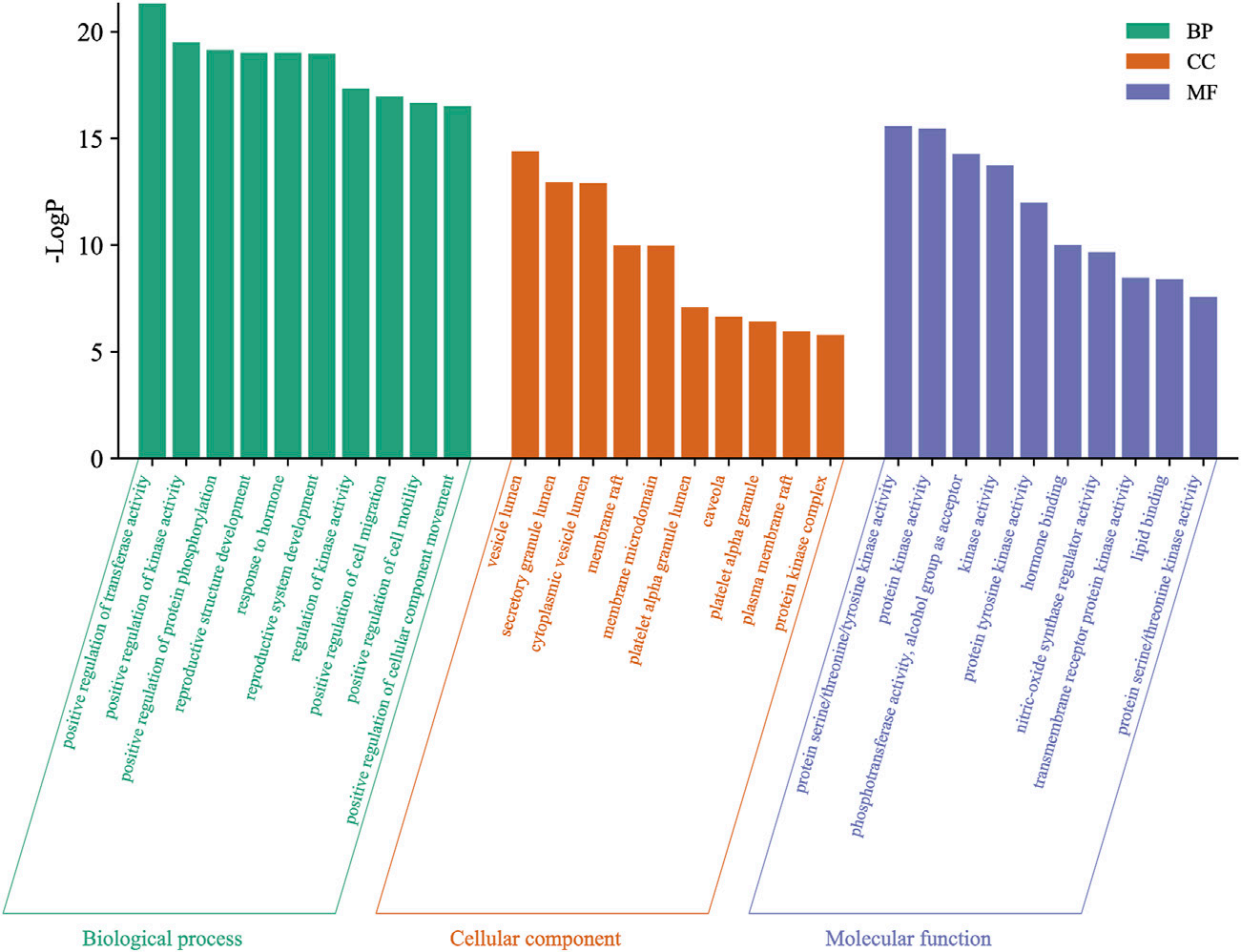
Visualization of Gene Ontology (GO) enrichment analysis. Biological process (BP), cellular component (CC), and molecular function (MF) of GO enrichment analysis are shown as green, orange, and purple bars, respectively.

GO enrichment analysis provided a systematic reflection of the mechanism of the four active compounds of MCE in the treatment of enteritis. A total of 749 BP, 47 CC, and 64 MF were obtained (Supplementary Table S4) via Metascape, and the top 10 enriched BP, CC, and MF are visualized and detailed in Figure 3. BP was primarily involved in the positive regulation of transferase and kinase activity, protein phosphorylation, positive regulation of cell motility, and response to hormone and reproductive system development. CC primarily included vesicle lumen, secretory granule lumen, and cytoplasmic vesicle lumen, whereas MF included protein serine/threonine/tyrosine kinase activity, protein kinase activity, phosphotransferase activity, nitric oxide synthase regulatory activity, and protein serine/threonine kinase activity.

**FIGURE 4:**
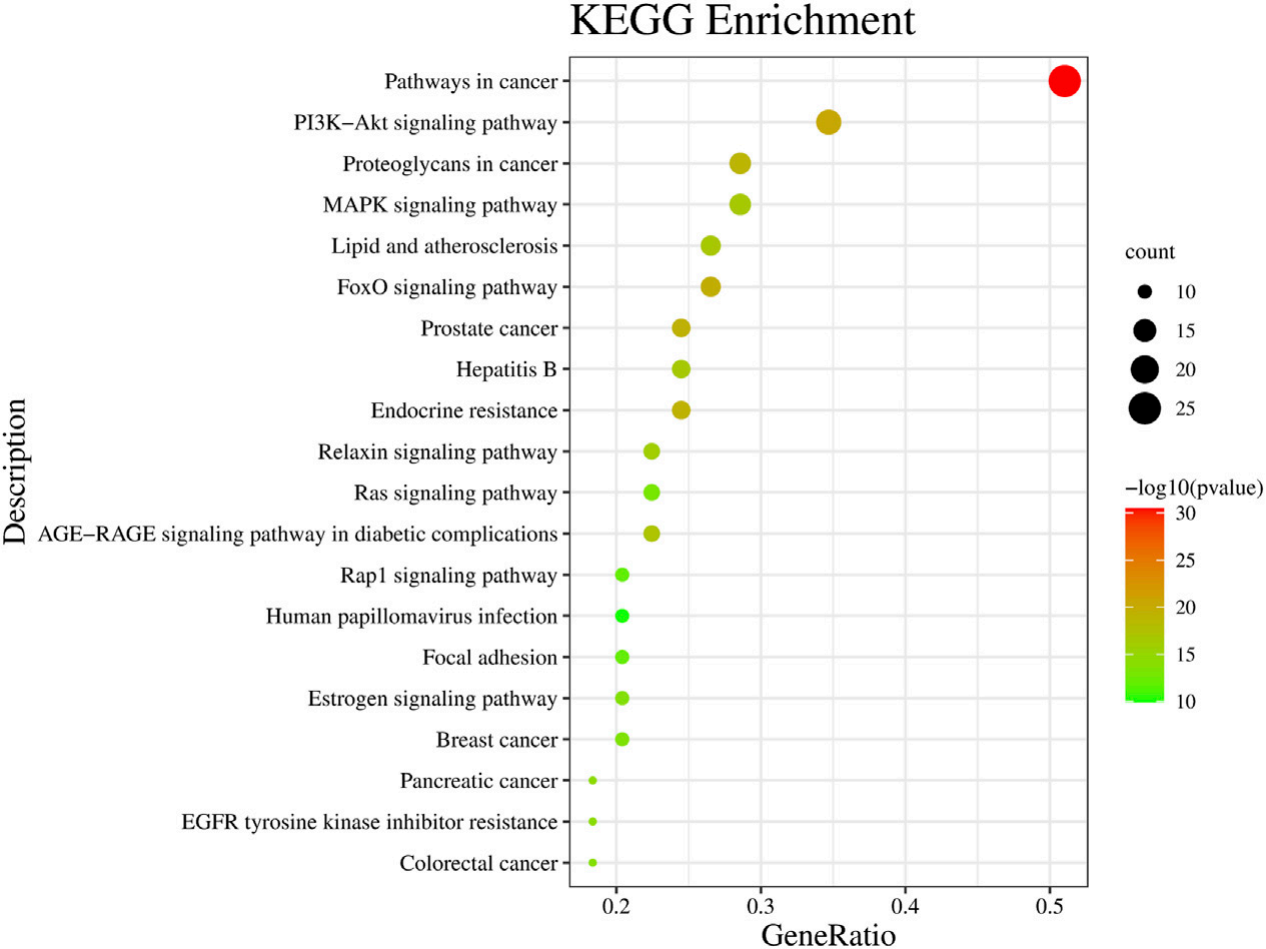
Kyoto Encyclopedia of Genes and Genomes (KEGG) signaling pathway enrichment analysis. Bubble color and size distribution represent −logP values and the number of genes involved in this signaling pathway, respectively.

The KEGG pathway analysis allows the prediction of the role of protein interaction networks in different cell activities. In this study, Metascape was used for KEGG pathway analysis, and 142 pathways were obtained (Supplementary Table S5), among which the top 20 enriched pathways are shown in Figure 4. The analysis showed that cancer-related pathways, PI3K-Akt signaling pathway, MAPK signaling pathway, FoxO signaling pathway, lipid and atherosclerosis, and Rap1 signaling pathway were mainly involved.

### 3.6 Drug–active compound–target–pathway–disease network construction

**FIGURE 5:**
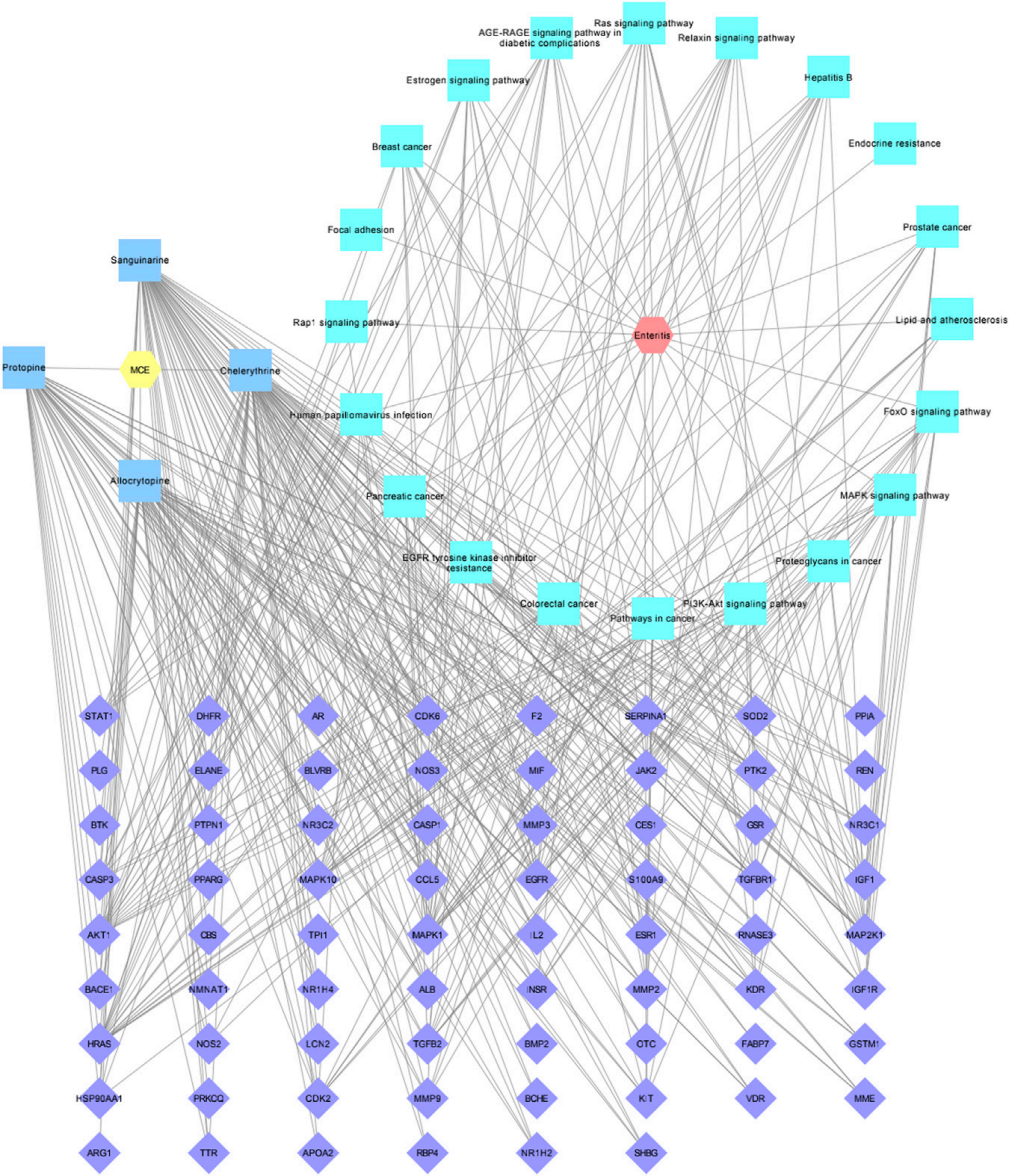
Drug–active compound–target–pathway–disease network diagram. The yellow hexagon represents MCE, the red hexagon represents enteritis, four blue squares represent MCE active compounds, 20 green squares represent pathways, and 70 purple diamonds represent intersection targets.

To visualize the potential molecular mechanism of the four active compounds of MCE in the treatment of enteritis, a “drug–active compound–target–pathway–disease” network diagram was built using Cytoscape 3.7.1 software. In the diagram, there are 96 nodes and 410 connections (Figure 5). Furthermore, 58 targets are connected to CHE, 55 to SAN, 44 each to ALL and PRO, 17 to the PI3K-Akt signaling pathway, 14 each to the cancer-related and MAPK signaling pathways, and 13 to the FoxO signaling pathway.

### 3.7 Molecular docking

**FIGURE 6:**
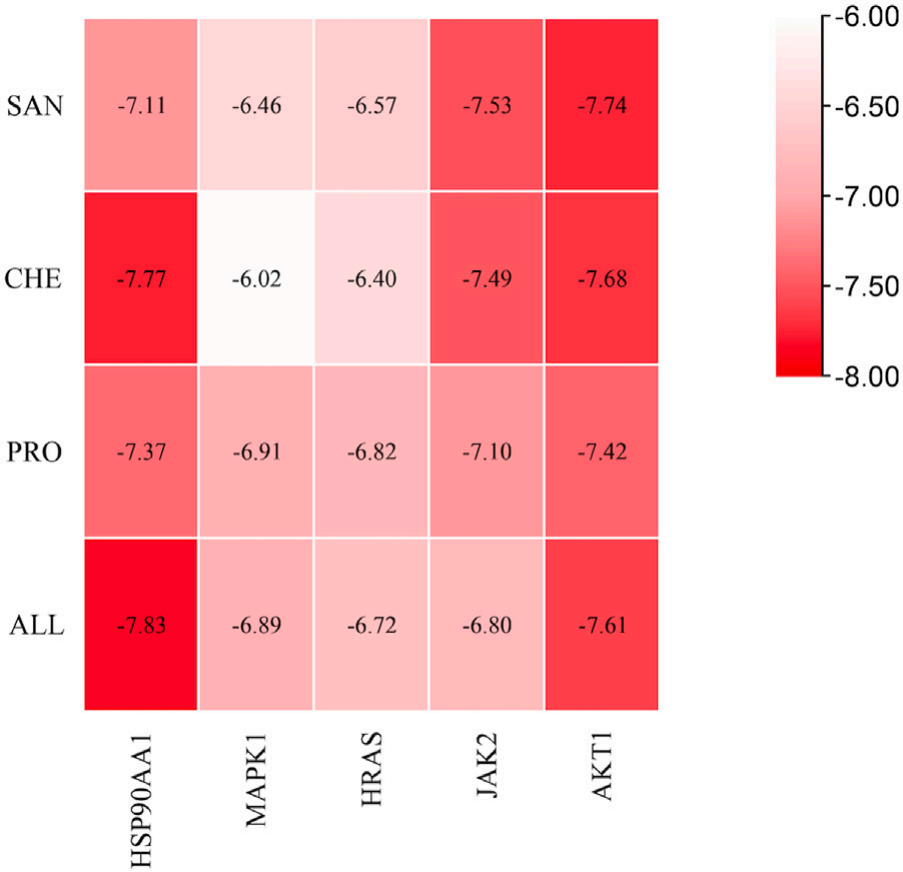
Heatmap of the molecular docking results between active compounds and core targets. The vertical coordinates represent the four active compounds of MCE, the horizontal coordinates represent the core targets, and the colors from red to white indicate the strongest to weakest binding abilities.

To determine the binding between small molecules of the drug and target macromolecules, binding energy was used: less than −5 kcal/mol indicates good affinity; the lower the binding energy, the stronger the affinity (Li et al., 2022). Molecular docking between the four active compounds of MCE and the five core targets was performed based on the results of the aforementioned analysis: the ligands were SAN, CHE, PRO, and ALL, and the receptors were HSP90AA1 (PDB ID: 7S9H), MAPK1 (PDB ID: 6G54), HRAS (PDB ID: 7JII), JAK2 (PDB ID: 7F7W), and AKT1 (PDB ID: 7NH5). The molecular docking binding energies were all less than −5 kcal/mol, indicating that the active compounds and the target had high affinity and could stably bind (Figure 6).

**FIGURE 7:**
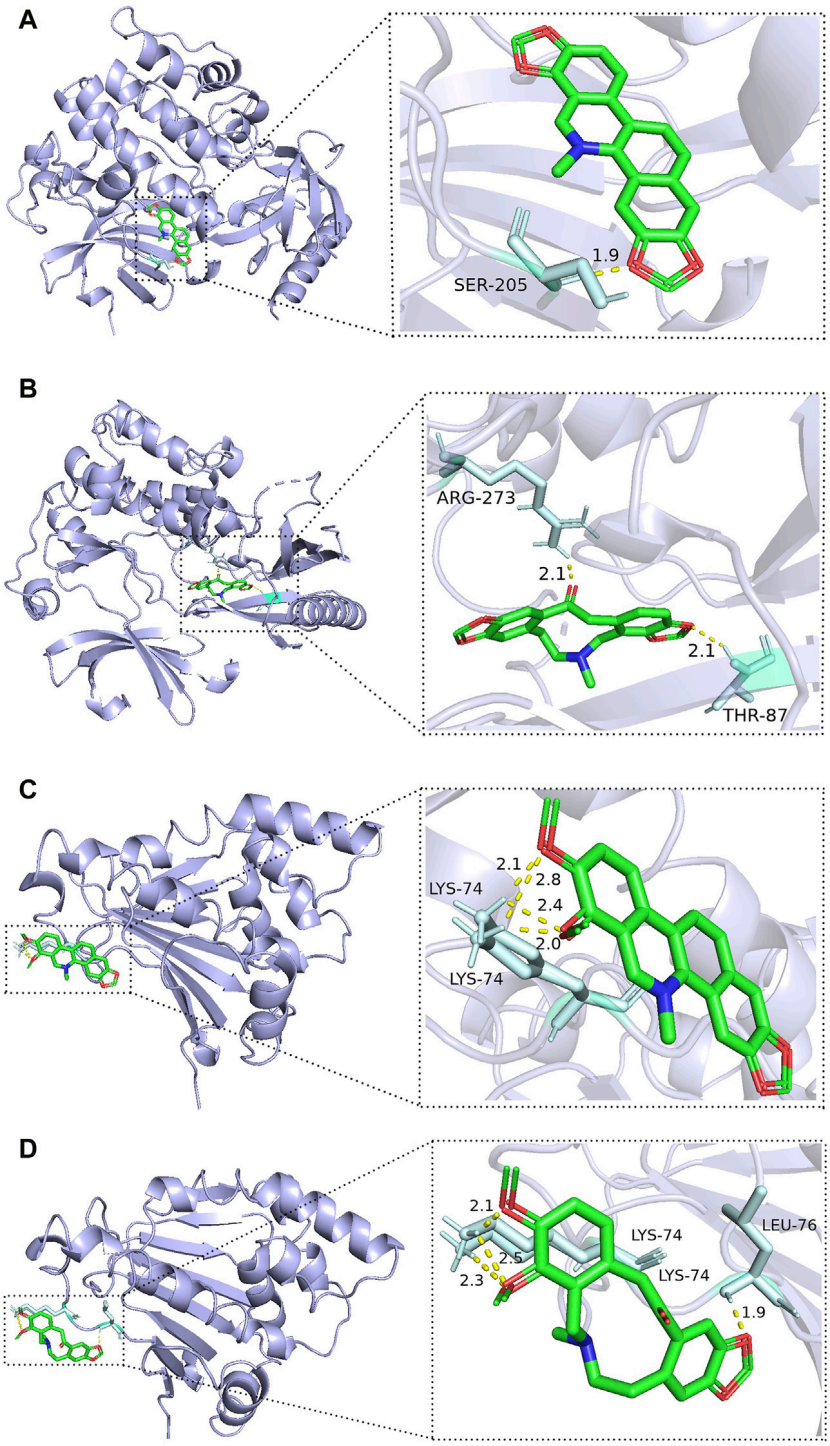
Best docking combinations for the four active compounds of MCE and core targets. The green model represents the MCE active compounds; the purple model represents the core targets, and the binding sites and binding distances of active compounds and target proteins are shown in the figure: **(A)** SAN–AKT1; **(B)** PRO–AKT1; **(C)** CHE–HSP90AA1; and **(D)** ALL–HSP90AA1.

The best binding results were obtained for SAN and PRO with AKT1 and for CHE and ALL with HSP90AA1 (Figures 7A–D). SAN formed one hydrogen bond with Ser 205 in AKT1, PRO formed one hydrogen bond with Arg 273 and one hydrogen bond with Thr 87 in AKT1, CHE formed four hydrogen bonds with two Lys 74 residues in HSP90AA1, and ALL formed one hydrogen bond with Leu 76 in HSP90AA1 and three hydrogen bonds with two Lys 74 residues.

## 4 Discussion

According to the literature, the primary constituents of MCE are isoquinoline alkaloids, which contain active compounds such as SAN, CHE, PRO, and ALL (Zeng et al., 2013; Ni et al., 2016; Lin et al., 2018). These compounds possess anti-inflammatory, antibacterial, insecticidal, anti-tumor, and intestinal health-improving functions. Wang et al. (2021) showed that MCE aids the increase in the height of intestinal villi and the decrease in crypt depth, thus improving nutrient utilization. It also increases the numbers of *Lactobacillus* and decreases those of *Escherichia*–*Shigella*, *Streptococcus*, *Proteobacteria*, and *Actinobacteriota*, thus ensuring optimal intestinal health. The four active compounds of MCE primarily act on AKT1 and MAPK1 to activate PI3K-Akt and MAPK signaling pathways for treating enteritis. MCE provides inflammation regulation through PI3K-Akt and MAPK signaling pathways and shows anti-inflammatory effects by inhibiting MAPK activation to alter the synthesis of inflammatory mediators (Dong et al., 2021; Zeng et al., 2022). These results are consistent with our network pharmacology prediction.

According to the network pharmacology analysis, there are 269 targets for the four active compounds of MCE, 1,237 targets for enteritis, and 70 targets for the intersection of drug and disease targets. The construction of the PPI network helped explore the core targets, and HSP90AA1, MAPK1, HRAS, JAK2, and AKT1 were obtained according to condition screening. HSPs are primarily induced by stressors, and the percentage of cellular proteins will elevate when the organism is subjected to microbial stimuli, such as parasites, fungi, viruses, or heat shock proteins (Zuehlke et al., 2015; Bolhassani and Agi, 2019; Shan et al., 2020). Most HSPs act as molecular chaperones for a variety of cellular processes in the organism (proper folding, activation, and assembly of proteins) (Li et al., 2019). The *HSP90AA1* gene encodes the heat shock protein 90α, a stress-inducible heterodimer of the molecular chaperone HSP90 (Zuehlke et al., 2015). The regulation of inflammatory cytokines expressed by HSP90, which is elevated when stimulated by inflammation and hypoxia, is primarily achieved through the activation of the NLRP3 inflammasome and cellular transcription factor NF-κB. This activity induces the activation of caspase-1 and the maturation and secretion of the downstream pro-inflammatory factors IL-1β and IL-18 (Sarvestani and McAuley, 2017; Li et al., 2019). MAPK1 is a significant MAPK signaling pathway component of the MAPK signaling pathway (Luo et al., 2020). This target component can regulate a variety of cellular physiological and pathological functions, such as cell growth, adhesion, and differentiation through transcription, translation, and cytoskeleton rearrangement, and it is a key signaling molecule in the treatment of inflammation-related diseases (Dong et al., 2021). HRAS activates the RAS/MAPK signaling pathway, which in turn regulates gene transcription and cell cycle (cell growth, division, maturation, and differentiation) (Kodaz et al., 2017). JAK2, a member of the intracellular tyrosine kinase family, is a potential target in the treatment of ulcerative colitis (Danese et al., 2019), as it regulates pathophysiological processes, such as inflammatory response, apoptosis, and cell cycle, primarily through its involvement in the JAK-STAT signaling pathway. JAK2 activates not only STAT3 but also ERK and p38 MAPK pathways (Truong et al., 2017), regulating innate and acquired immunity and reducing chronic inflammation in the intestine (Zhao et al., 2021). AKT1, a serine/threonine protein kinase and one of the key downstream regulatory proteins of PI3K, is involved in a variety of cellular processes *in vivo*, such as cell proliferation, metabolism, apoptosis, and angiogenesis, using serine and/or threonine phosphorylation of downstream substrates (Hers et al., 2011). AKT1, the major isoform of Akt in endothelial cells, plays a key role in causing cardiovascular diseases, where the pathogenesis of atherosclerosis is closely linked to the inflammatory response (Chen et al., 2005; Fougerat et al., 2009; Hers et al., 2011). Thus, the four active compounds of MCE can achieve a multi-component and multi-targeted treatment for enteritis by acting on the core targets.

GO and KEGG enrichment analyses are helpful in exploring the signaling pathway of the four active compounds of MCE in the treatment of enteritis. According to GO enrichment analysis, the four active compounds of MCE helped in the treatment of enteritis primarily through the involvement of transferase and kinase activity, protein phosphorylation, and cell motility. In addition, cancer-related pathways, PI3K–Akt signaling pathway, MAPK signaling pathway, FoxO signaling pathway, and Rap1 signaling pathway play a vital role in the treatment of enteritis using the four active compounds of MCE. Because the PI3K-Akt signaling pathway enriched five core targets, it could be the main signaling pathway for processing enteritis. The PI3K-Akt signaling pathway is the main pathway in the regulation of inflammation, primarily in the regulation of inflammatory cell recruitment, expression, and activation of inflammatory mediators (Fougerat et al., 2009; Huang et al., 2011). This pathway can also promote inflammation by activating NF-κB to cause the release of inflammatory factors (IL-6 and IL-8), thereby sustaining and amplifying the inflammatory response (Huang et al., 2011). Fang et al. (2018) showed that berberine ameliorates necrotizing enterocolitis through the PI3K-Akt signaling pathway. Yan et al. (2022) showed that Huang Bai Jian Pi decoction relieves diarrhea and regulates the expression of inflammatory factors through the PI3K-Akt signaling pathway. These results suggest that the PI3K-Akt signaling pathway plays a regulatory role in enteritis, which is consistent with our network pharmacological prediction. The MAPK signaling pathway is involved in various biological processes such as immunity, stress, cell growth, apoptosis, and differentiation, and it plays a key role in the release of pro-inflammatory cytokines (Luo et al., 2020; Afroz et al., 2021). The MAPK family includes three subfamilies, namely, ERK, JNK, and p38 MAPK, which are associated with intestinal mucosal injury and are activated in inflammatory bowel disease to induce the release of cytokines such as IL-8 (Luo et al., 2020). Dong et al. (2020a) reported that *Astragalus* polysaccharide could alleviate lipopolysaccharide (LPS)-induced intestinal inflammation via the MAPK signaling pathway. Dong et al. (2020b) showed that oleanolic acid maintained the intestinal barrier, improved *Salmonella typhimurium*-induced diarrhea in mice, and reduced intestinal inflammation through the MAPK signaling pathway. Therefore, the MAPK signaling pathway plays a regulatory role in enteritis, a result consistent with our network pharmacological prediction. Thus, it is evident that the four active compounds of MCE possess pharmacological effects in the treatment of enteritis through multiple pathways.

Based on the aforementioned network pharmacological analysis, four active compounds, SAN, CHE, PRO, and ALL, are stably bound to five core targets, HSP90AA1, MAPK1, HRAS, JAK2, and AKT1, which was validated using molecular docking. Thus, the interaction between the active compounds and the target may be one of the most important pharmacological mechanisms of the four active compounds of MCE in the treatment of enteritis, an outcome that needs to be verified by further experiments.

However, the potential pharmacological mechanism of the four active compounds of MCE in the treatment of enteritis was explored using only network pharmacology and molecular docking techniques. In addition, the current network information is not comprehensive, and the real-time data are not available in the databases. Therefore, the findings of this study need to be further validated in animals for better clinical applications.

## 5 Conclusion

The four main active compounds of MCE involved in the treatment of enteritis are SAN, CHE, PRO, and ALL, and the five core targets are HSP90AA1, MAPK1, HRAS, JAK2, and AKT1. PI3K-Akt and MAPK, obtained through network pharmacology and molecular docking technologies, are the main signaling pathways. These results are of great significance for further exploration of the potential pharmacological mechanism of MCE in the treatment of enteritis.

## Data Availability

Publicly available datasets were analyzed in this study. The names of the repository/repositories and accession number(s) can be found in the article/Supplementary Material, further inquiries can be directed to the corresponding author.
