# Supplementary material for: Potential pharmacological mechanisms of four active compounds of Macleaya cordata extract against enteritis based on network pharmacology and molecular docking technology
Source: Front Physiol. 2023 May 2;14:1175227. doi: 10.3389/fphys.2023.1175227 (PMC10185776; doi:10.3389/fphys.2023.1175227)
Supplement: Supplementary file 6 [file Image1.pdf]

*Supplementary Material*

# Potential pharmacological mechanisms of four active compounds of *Macleaya cordata* extract against enteritis based on network pharmacology and molecular docking technology

**Pingrui Yang[1,†], Chonghua Zhong[2,3,†], Huan Huang[1], Xifeng Li[1], Lin Du[1], Lifang Zhang[1], Shicheng Bi[1,4], Hongxu Du[1,4], Qi Ma[1,4] and Liting Cao[1,4*]**

*** Correspondence: Corresponding Author: (L.T. Cao) caoliting@swu.edu.cn**

## 1 Supplementary Figures

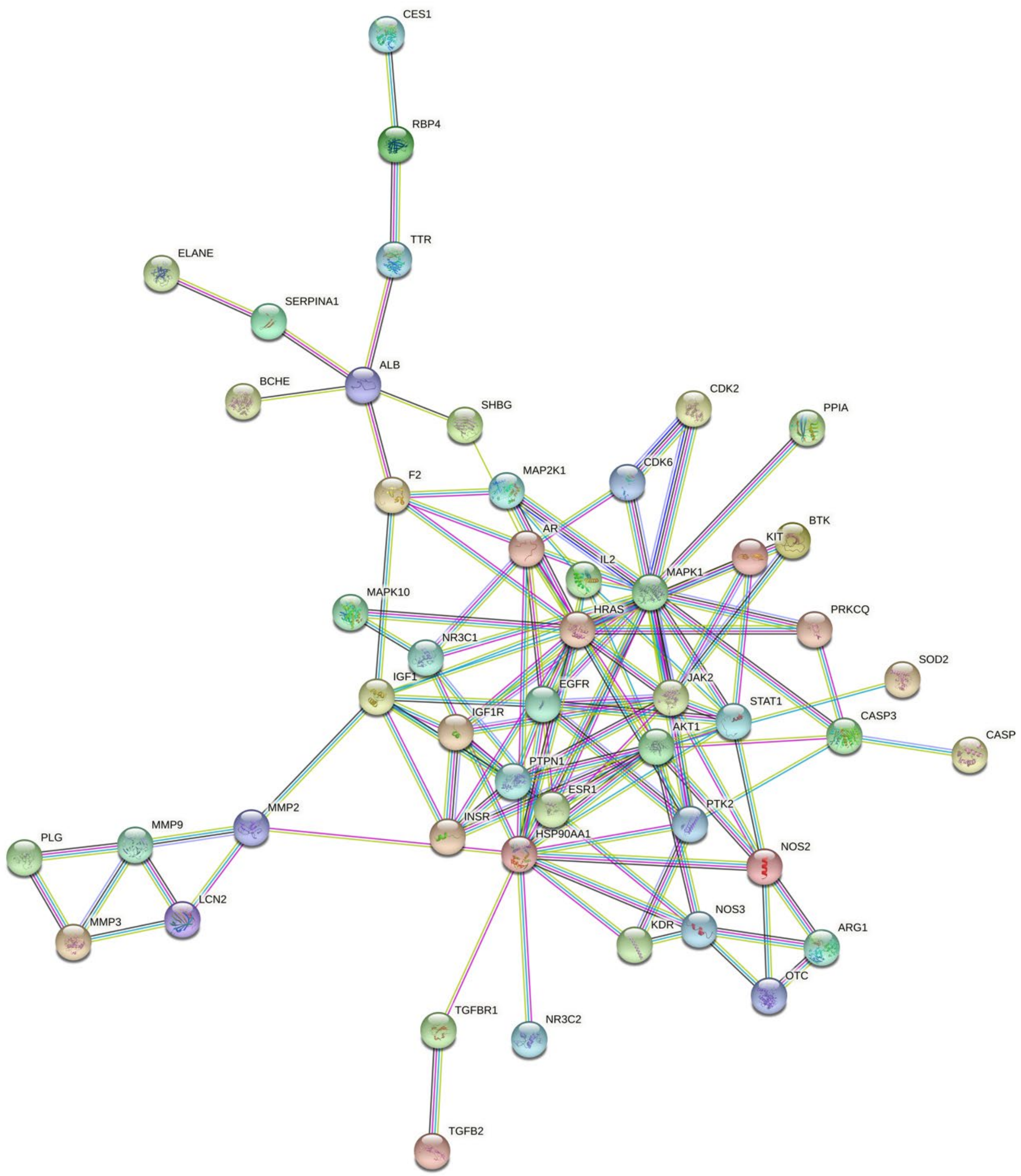


**Supplementary Figure S1.** Protein-protein interaction (PPI) network constructed by STRING. It represents the interaction target of active compounds and enteritis.
